# Homotypic and heterotypic *in cis* associations of MHC class I molecules at the cell surface

**DOI:** 10.1016/j.crimmu.2022.05.001

**Published:** 2022-05-23

**Authors:** Fernando M. Ruggiero, Sebastian Springer

**Affiliations:** School of Science, Jacobs University Bremen, Campus Ring 1, D-28759, Bremen, Germany

**Keywords:** MHC class I, Dimers, Oligomers, Free heavy chain, Homotypic associations, Heterotypic associations, Open conformers, Closed conformers, Empty conformers, Non-covalent associations

## Abstract

Through the presentation of peptide antigens to cytotoxic T lymphocytes, major histocompatibility complex (MHC) class I molecules mediate the adaptive immune response against tumors and viruses. Additional non-immunological functions include the heterotypic association of class I molecules with cell surface receptors, regulating their activities by unknown mechanisms. Also, homotypic associations resulting in class I dimers and oligomers - of unknown function - have been related to pathological outcomes. In this review, we provide an overview of the current knowledge about the occurrence, biochemical nature, and dynamics of homotypic and heterotypic associations of class I molecules at the cell surface with special focus on the molecular species that take part in the complexes and on the evidence that supports novel biological roles for class I molecules. We show that both heterotypic and homotypic class I associations reported in the literature describe not one but several kinds of oligomers with distinctive stoichiometry and biochemical properties.

## Highlights

• Major histocompatibility complex class I molecules form homotypic and heterotypic associations at the cell surface.
• Associations show distinctive stoichiometry and biochemical properties.
• Associations might regulate immunological and non-immunological processes.
• Heterotypic association with cell surface receptors might regulate receptor's activity.
• Homotypic associations have been related to pathological outcomes.

## Graphical abstract

**Figure:**
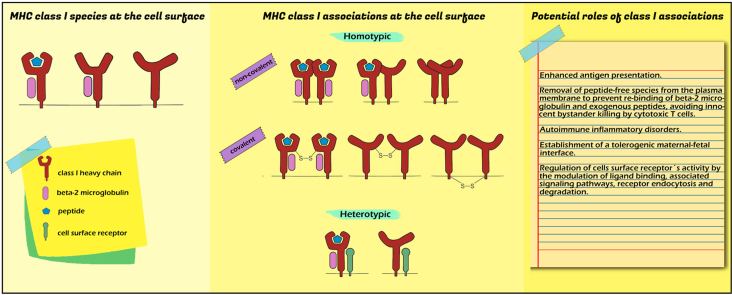

## 1 Introduction

Major histocompatibility (MHC) class I molecules mediate the cellular adaptive immune response by presenting antigenic peptides of virus-infected and tumorigenic cells to cytotoxic CD8^+^ lymphocytes (Townsend and Bodmer, 1989; Huppa and Davis, 2003). Besides this classically recognized function, other immunological roles exist: for natural killer (NK) cells, MHC class I molecules are inhibitory ligands; thus, cells that lack one or more self-MHC class I molecules on their surfaces may be rapidly eliminated as the result of NK cell activation (missing self hypothesis) (Ljunggren, 2021; Ljunggren and K ä rre, 1990).

Recently, it has become evident that class I molecules are involved in non-immunological tasks, regulating synaptic remodeling and plasticity (Cebrián et al., 2014; Shatz, 2009) and synapse density in the developing brain (Glynn et al., 2011; Elmer et al., 2013). Roles in neurological and psychiatric disorders such as amyotrophic lateral sclerosis, schizophrenia, and bipolar disorder have been described (Stefansson et al., 2009; Shi et al., 2009). Such roles might involve the modulation of intracellular signal transduction events through the association of class I molecules with cell surface receptors on the same cell (*in cis* heterotypic associations) (Dixon-Salazar et al., 2014; Fishman et al., 2004). However, the molecular mechanisms by which class I molecules might regulate the receptor activity are not well understood.

There is also evidence for *in cis* homotypic interactions, *i.e.*, associations of class I molecules with other class I molecules at the cell surface. Such homotypic associations might be involved in immunological or non-immunological processes. For example, oligomers of class I free heavy chains observed on the cell surface of neurons were found to negatively regulate synapse density during the establishment of neuronal connections (Glynn et al., 2011; Elmer et al., 2013). In addition, covalent HLA-B*27:05 dimers were postulated to be ligands of activating NK cell receptors and to trigger autoimmune inflammatory disease (Chen et al., 2017). The molecular mechanisms of such processes warrant further investigation.

**Fig. 1:**
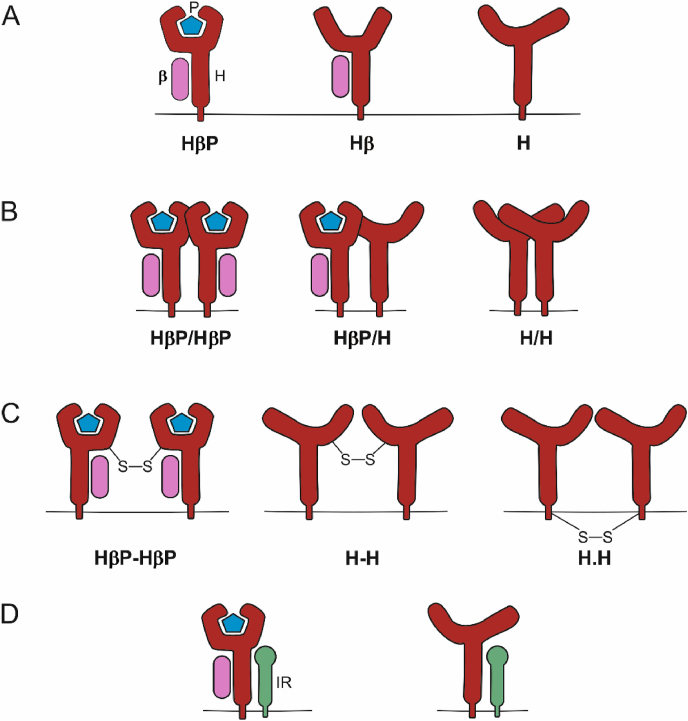
**Known species of MHC class I monomers and dimers. *A*,** Schematic representation of the different molecular species of class I molecules (HβP, Hβ and H) found at the cell surface. P: antigenic peptide; H: class I transmembrane heavy chain; β: beta-2 microglobulin. ***B*,** Non-covalent homotypic associations between class I species (HβP/HβP, HβP/H and H/H) of the same or different allotypes (Table 1). ***C*,** Covalently bound class I dimers. For those dimers formed between free heavy chains (H), the same or different allotypes might be involved, and the disulfide bond links the extracellular domains (H-H) or the cytosolic domains (H**.**H). Covalent dimers of HβP species (HβP**-**HβP) were only reported for HLA-G (Table 1). ***D*,** Example of heterotypic association. The non-covalent interaction between HβP or H with the insulin receptor (IR) is depicted as an example. The structure of the IR is simplified and does not reflect the actual structure of the receptor. Different interacting partners were described in the literature (Table 2). For clarity, only dimeric associations of a single allotype are shown.

There are three forms, or species, of monomeric class I molecules at the cell surface (Fig. 1A). Antigenic peptides (P) are presented to cytotoxic T cells as part of a non-covalent complex formed by the polymorphic class I transmembrane heavy chain (H) and the non-polymorphic light chain beta-2 microglobulin (β_2_m, β). This heavy chain/β_2_m/peptide (HβP) complex is assembled in the endoplasmic reticulum (ER) and then exported to the plasma membrane (Fritzsche and Springer, 2013; Donaldson and Williams, 2009; Peaper and Cresswell, 2008), where it typically resides for hours to days (Springer, 2015; Lu et al., 2012).

Peptide-free class I molecules are also known to exist. These can be either complexes between heavy chain (H) and β_2_m (*i.e.*, Hβ) or else free heavy chains (H) (Fig. 1A). Hβ may escape from the ER before loading with high-affinity peptide (Montealegre et al., 2015; Ortiz-Navarrete and Hämmerling, 1991; Day et al., 1995; Saini et al., 2013; Allen et al., 1986a), and/or they may arise after dissociation of the peptide from HβP at the cell surface or in recycling endosomes (Montealegre et al., 2015; Dirscherl et al., 2018; Matko et al., 1994; Rock et al., 1991a; Schumacher et al., 1990; Hochman et al., 1991; Pickl et al., 1996). Some Hβ can rapidly re-bind peptides to form HβP (Sugita and Brenner, 1994; Saini et al., 2019), whereas others cannot (Montealegre et al., 2015). The steady state level of Hβ remains low compared to HβP, since Hβ are rapidly internalized from the cell surface by endocytosis (Montealegre et al., 2015; Merzougui et al., 2011; Montealegre and van Endert, 2018). Alternatively, Hβ dissociate, releasing β_2_m as a soluble protein, with the resulting H remaining membrane-associated (Dirscherl et al., 2018). If the loss of β_2_m from Hβ is delayed by mutation (or in a recombinant single-chain Hβ construct), then cell surface removal is dramatically slowed, suggesting that endocytic degradation occurs via H (Montealegre et al., 2015).

As mentioned, H are formed at the plasma membrane from Hβ, but they also exist in the ER and Golgi complex of β_2_m-defective, virus-infected, and tumorigenic cells, from where they can travel through the secretory pathway to reach the plasma membrane (Lu et al., 2012; Capps et al., 1993; Potter et al., 1984; Lhotakova et al., 2019). There are two biochemically different species of H, one that is able to re-bind β_2_m, producing Hβ (Chakrabarti et al., 1992), whereas the other one (perhaps as a result of a slow conformational rearrangement of the former species) cannot re-associate with β_2_m and peptide and thus remains as H (Montealegre et al., 2015; Matko et al., 1994; Edidin et al., 1997; Marozzi et al., 1993).

For both Hβ and H, the experimental observations of a short lifetime and a low steady-state level might – at least partially – be caused by their participation in homotypic and/or heterotypic associations that conceal epitopes, precluding their detection by antibodies (Matko et al., 1994). This is in line with the many observations that report large amounts of Hβ at the cell surface as shown by addition of exogenous peptides (Ortiz-Navarrete and Hämmerling, 1991; Day et al., 1995; Saini et al., 2013; Schumacher et al., 1990; Sugita and Brenner, 1994; Ljunggren et al., 1990; Christinck et al., 1991; Neefjes et al., 1992; Burshtyn and Barber, 1993; Carreno and Hansen, 1994; Su et al., 1998). Thus, the observed short lifetime of Hβ and H does not necessarily argue against a biological function for these molecules. Indeed, major efforts have been undertaken to uncover the occurrence and the roles of homotypic and heterotypic associations involving all three different species of class I molecules, as described in the following sections.

In the literature, the different class I molecular species are usually referred to as “closed” (corresponding to HβP) and “open” or even “empty” (corresponding to Hβ and/or H) conformers. But these terms do not provide a precise molecular description. The term “empty” is particularly inaccurate, since the lack of bound peptide does not necessarily imply that the binding groove is empty. Indeed, a recent study showed that the peptide binding groove of HLA-A2 was occupied by small organic molecules when it was in a peptide-free form (Anjanappa et al., 2020). Water molecules in the binding groove might also contribute to the binding energy of peptides, playing an active role as mediators in the MHC-peptide interaction (Petrone and Garcia, 2004). Hβ also bind dipeptides and short peptides (Saini et al., 2013, 2015; Ljunggren et al., 1990) or low-affinity full-length peptides. Due to weak binding and rapid dissociation of these peptides, they cannot be co-isolated with class I upon cell lysis and immunoprecipitation. Thus, the word “empty” is inaccurate, and Hβ that can bind peptide are better called peptide-receptive. Finally, the term “open conformers” alludes to the venus flytrap structural model of peptide binding, in which the lateral helices of the peptide binding groove are hypothesized to open up outwards, away from each other, when no peptide is bound. In contrast to this simplistic static model, it is now known that the main property of peptide-empty class I molecules is the conformational fluctuation, or instability, of the peptide binding groove, which strongly depends on the allotype, and which may lead – on the average of conformational fluctuation – to an inward, outward, or no net movement of the helices (Jantz-Naeem and Springer, 2021; Bouvier and Wiley, 1998; Kurimoto et al., 2013; Zacharias and Springer, 2004). Also, the term “open conformers” does not differentiate the Hβ and H species, which have very distinct biochemical properties (Montealegre et al., 2015).

In this review, we describe the molecular species of class I that are involved in homotypic and heterotypic associations at the cell surface (Table 1, Table 2). Table 3, Table 4 show the class I allotypes and the specificity of the antibodies in each experimental approach. Literature information was sometimes insufficient to precisely assess the molecular species involved in the associations.

**Table 1:** Some reported homotypic *in cis* interactions between class I molecules.

Allotype	Species in the complex	Homotypic association	Cells, mice, model	Antibody	Methodology	Ref.
HLA-A2, B7, C7	H	Oligomers 3	JY cells	HC-10	FRET by flow cytometry	Matko et al. (1994)

HLA-A2, B7, C7	HβP 1 and H		JY cells	KE-2	EEQ by flow cytometry	

HLA-A, B, C	HβP 1 and H	Activated B or T-cells	KE-2	EEQ by flow cytometry

HLA-A2	HβP 1 and H	Between HLA-A2	JY cells	BB7.2	FRET by flow cytometry	Chakrabarti et al. (1992)

HLA-A2	HβP 1 and H	Affinity purified (BB7.2) and labelled HLA-A2 reconstituted in liposomes	–	FRET by flow cytometry, TPA, FRAP

**H-2L**^**d**^	H	Dimers 4	HCT-Ld3 cells	28.14.8, 64.3.7	Cell surface labeling using^125^I or metabolic labeling followed by IP	Capps et al. (1993)

H-2D^b^, H-2L^d^	H	Dimers c^,^^4^	Eld3 cells	28.14.8		

H-2^d^	HβP, Hβ 2, H 2	H-2L^d^ dimers 4 and oligomers	BALB/c splenocytes	28.14.8		

H-2L^d^	H	H-2L^d^ dimers 4	BALB/c splenocytes	64.3.7

H-2^b^	HβP, Hβ 2, H 2	H-2D^b^ dimers 4 and oligomers	C57BL/6 splenocytes	28.14.8

H-2D^d^	H, Hβ 2	Dimers 4	18.48 cells	34.2.12

**H-2K**^**b**^	H	Non-covalent dimers	STF1 cells transfected with H-2K^b^	–	Two-hybrid assay based on antibody micropatterns	Dirscherl et al. (2018)

**H-2K**^**b**^	HβP	Between K^b^ bound to OVA peptide SIINFEKL	_L_-K^b^ cells	25-D1.16	TIRFM	Lu et al. (2012)

**H-2K**^**b**^	HβP	Non-covalent dimers mediated by N-linked sugars	Extracellular domain of H-2K^b^ fused to His tag and captured by Nickel-chelating lipid in artificial bilayers	–	Two-dimensional crystallization followed by electron microscopy and molecular replacement calculations	Mitra et al. (2004)

HLA-A2, B7, C7, C4	HβP and H	Dimers and trimers 3	Raji cells	W6/32	Cell surface biotinylation or metabolic labeling followed by IP	Triantafilou et al. (2000)

HLA-A2, B7, C7	HβP 1 and H	Dimers and trimers 3	JY cells	W6/32, KE-2, HC-10	SNOM, FRET by flow cytometry, pbFRET	Bodnar et al. (2003)

H-2K^b^	HβP	Oligomers	EL4 cells	AF6-88.5	TEM	Ferez et al. (2014)

H-2D^b^	HβP	Oligomers	Dendritic cells	B22.249		

HLA-A9, A25, B7, B41	HβP	Oligomers 3	Jurkat cells	W6/32

**HLA-A2 (YFP)**	HβP, Hβ 2, H 2	Between HLA-A2-YFP with itself or with endogenously expressed A2, B7 or C7	JY cells	–	TIRFM	Fooksman et al. (2006)

HLA-A2, B7, C7	HβP	Each allotype with itself or with any other allotype 3	JY cells	W6/32	AFM, TEM, FRET by flow cytometry	Jenei et al. (1997)

HLA-A66	HβP	Between HβP of HLA-A66	HUT-102B2 cells	W6/32

HLA-A2, B7, C7	HβP	Each allotype with itself or with any other allotype 3	JY cells	W6/32, KE-2, HC-10	FRET by flow cytometry, pbFRET	Bodn á r et al. (1996)

HLA-B27	H	Dimers 4^,^^5^ through Cys-67	–	HC-10	In vitro refolding followed by R SDS-PAGE and SEC	Allen et al. (1999)

HLA-B27	H	Tetramers	T2 cells transfected with HLA-B27	W6/32 (IP) HC-10 (blot)	Cell surface biotinylation followed by IP and NR SDS-PAGE

HLA-A2, B7, C7	HβP, Hβ 2, H 2	Each allotype with itself or with any other allotype 3	JY cells	–	Cross-linked MS	Armony et al. (2021)

HLA-A2, B27, C1	H	Dimers 3	Jesthom cells	HC-10	NR SDS-PAGE	Lynch et al., (2009)

HLA-A2, B27, C1	H	Dimers 3^,^^4^	Jesthom exosomes	HC-10	NR/R SDS-PAGE, NR 2D electrophoresis	Lynch et al. (2009)

HLA-A2	H	Dimers of HLA-A2	Jesthom exosomes	HCA2	NR 2D electrophoresis	

HLA-A2, B27, C1	H	Dimers 3	Jesthom exosomes	HC-10, HCA2	NR 2D electrophoresis	

HLA-A2, B27, C1	HβP, H	HβP/H Dimers 3	Jesthom exosomes	W6/32 (IP), HC-10 (blot)	IP followed by NR SDS-PAGE	

HLA-A2, B27, C1	HβP, H	Dimers of HLA-B27 HβP with H of itself or H of any other allotype 3	Jesthom exosomes	ME1 (IP), HC-10 (blot)	IP followed by NR SDS-PAGE	

HLA-A2	HβP, H	HβP/H dimers of HLA-A2	Jesthom exosomes	BB7.2 (IP), HCA2 (blot)	IP followed by NR SDS-PAGE	

HLA-A2, B27, C1	HβP, H	Dimers between HβP of HLA-A2 and H of any other allotype 3	Jesthom exosomes	BB7.2 (IP), HC-10 (blot)	IP followed by NR SDS-PAGE	

**HLA-B27**	H	Dimers of HLA-B27	Exosomes of C58 cells transfected with HLA-B27	HC-10	NR SDS-PAGE

**HLA-A2**	H	Dimers 5 of HLA-A2 that are no longer detected when a cytoplasmic domain-deleted “tail-less” version is expressed	Exosomes of C58 cells transfected with wild-type or cytoplasmic domain-deleted “tail-less” HLA-A2	HCA2	NR SDS-PAGE

HLA-A30, A31, A33, B35, **B27**	H	Dimers detected in B27 transfected cells. In non-transfected cells, dimers are undetectable	Exosomes of KG-1 cells transfected with HLA-B27	HC-10	NR SDS-PAGE

HLA-A30, A31, A33	H	Dimers 3	KG-1 cells	HCA2	NR SDS-PAGE

**HLA-B27**	H	Dimers 5 of HLA-B27 through Cys-325	Exosomes of LCL 721.221 cells transfected with wild-type, C308A or C325A HLA-B27	HC-10	NR SDS-PAGE

HLA-A2, B27, C1	H	Dimers of endogenously expressed allotypes only detectable after diamide treatment	Jesthom cells	HC-10	NR SDS-PAGE

HLA-A2, B27, C1	HβP	Dimers of endogenously expressed allotypes only detectable after diamide treatment	Jesthom cells	W6/32	IP followed by NR SDS-PAGE

HLA-A1, A31, B08, B40, **B27**	H	Dimers 5 detected in HLA-B27 transfected cells and only after diamide treatment. In non-transfected cells, dimers were undetectable even after diamide treatment	CEM cells transfected with HLA-B27	HC-10	NR SDS-PAGE	Makhadiyeva et al. (2012)

**HLA-B27**	H	Dimers 5 of HLA-B27 through Cys-325	Exosomes of LCL 721.221 cells transfected with wild-type, C308A or C325A HLA-B27	HC-10		

**HLA-A2**	H	Dimers 5 of HLA-A2 that are no longer detected when a cytoplasmic domain-deleted “tail-less” version is expressed	Exosomes of C58 cells transfected with wild-type or cytoplasmic domain-deleted “tail-less” HLA-A2	HCA2

**HLA-B7**	H	Dimers 4^,^^5^ of HLA-B7 through Cys-325 and Cys-308	LCL 721.221 cells transfected with HLA-B7	HC-10	NR/R SDS-PAGE, mutagenesis	Baia et al. (2016)

**HLA-B27**	H	Dimers of HLA-B27	LCL 721.221 cells transfected with HLA-B27	HC-10	NR SDS-PAGE	

**HLA-A2**	H	Dimers of HLA-A2	LCL 721.221 cells transfected with HLA-A2	HCA2	NR SDS-PAGE

**HLA-A3**	H	Dimers of HLA-A3	LCL 721.221 cells transfected with HLA-A3	HCA2	NR SDS-PAGE

**HLA-G**	HβP	Dimers 4 of HLA-G through Cys-42	LCL 721.221 cells transfected with HLA-G	BBM.1	Cell surface biotinylation followed by IP and NR/R SDS-PAGE	Boyson et al. (2002)

**HLA-A2**	HβP	Dimers 4 of HLA-A2. Addition of iodoacetamide abrogated dimer formation, demonstrating that these are artifactual dimers	LCL 721.221 cells transfected with HLA- A2	BBM.1

**HLA-G**	HβP	Dimers 4^,^^5^ through Cys-42, trimers 4^,^^5^ through Cys-42, and Cys-147, and oligomers	LCL 721.221 cells transfected with HLA-G	MEM-G/09 (IP)	Cell surface biotinylation followed by IP and 2D (NR/R) SDS-PAGE	Gonen-Gross et al. (2003)

HLA-G	HβP	Dimers 4^,^^5^, trimers, and oligomers	Jeg-3 cells	MEM-G/09 (IP)	Cell surface biotinylation followed by IP and 2D (NR/R) SDS-PAGE	Gonen-Gross et al. (2005)

HLA-G	HβP	Dimers through Cys-42	–	–	In vitro refolding followed by crystallization	Shiroishi et al. (2006)

HLA-G	HβP	Dimers	Human-derived first-trimester trophoblast cells	MEM-G/11 (IP), 87G (IP)	Cell surface biotinylation followed by IP and NR/R SDS-PAGE	Apps et al. (2007)
HLA-G	HβP	Dimers		G233 (IP)		

**Abbreviations**: AFM: atomic force microscopy; APCs: antigen presenting cells; CTL: cytotoxic T lymphocytes; EEQ: electron-exchange quenching; FRAP: fluorescence recovery after photobleaching; FRET: Förster resonance energy transfer; pFRET: photobleaching FRET; IP: immunoprecipitation; MS: mass spectrometry; NR SDS-PAGE: non-reducing SDS-polyacrylamide gel electrophoresis; R SDS-PAGE: reducing SDS-PAGE; SEC: size exclusion chromatography; SNOM: scanning near field optical microscopy; TEM: transmission electron microscopy; TIRFM: total internal reflection fluorescence microscopy; TPA: time-resolved phosphorescence anisotropy.

**Notes:** First column shows endogenously expressed (not bold) and transfected (bold) allotypes by each cell line. A list of the allotypes expressed by cells and mice, the binding specificity, and the class I species (HβP, Hβ or H) that are recognized by antibodies can be found in Table 3, Table 4, respectively.

1 HβP associations that were only detected when HC-10 reactive class I molecules (H) were also present, or HβP associations that were reduced after addition of exogenous β_2_m.

2 Uncertainty about the class I molecular species (HβP, Hβ and/or H) involved in the homotypic association.

3 Each allotype with itself or with other allotypes. Not possible to unequivocally define which of the allotypes took part in the described association.

4 Dimers sensitive to reducing agents.

5 Covalent dimers confirmed by mutagenesis.

**Table 2:** Some heterotypic *in cis* interactions of class I molecules from the literature.

Allotype	Species in the complex	Associated Partner	Cells, mice, model	Antibody	Methodology	Ref.
H-2D^b^, H-2K^b^	HβP	Insulin receptor	C57BL/6J mice	20.8.4	Cell surface labeling of IR with^125^I-photoreactive insulin analog followed by IP and SDS-PAGE	Chvatchko et al. (1983)

H-2K^k^, H-2D^k^	HβP	AKR/J mice	3.83

H-2K^k^	HβP	Insulin receptor 3	C3H mice	H-100-30/23, 3.83, H-100-5/28, 11.4.1, 16.3.1	Cell surface labeling of IR with^125^I-photoreactive insulin analog followed by IP and SDS-PAGE	Fehlmann et al. (1985)

H-2D^k^	HβP		C3H mice	H-100-30/23, 3.83		

H-2K^b^, H-2D^b^	HβP	C57BL/6J mice	20.8.4

**H-2D**^**b**^	HβP, Hβ 2, H	Insulin receptor	RE1 cells transfected with β_2_m and H-2D^b^	28.14.8	Cell surface labeling of IR with^125^I-photoreactive insulin analog followed by IP and SDS-PAGE	Verland et al. (1989)

H-2K^k^	HβP	Insulin receptor	C3H mice	11.4.1	Cell surface labeling of IR with^125^I-photoreactive insulin analog followed by IP and SDS-PAGE	Phillips et al. (1986)

H-2D^k^	2		C3H mice	15.5.5S		

H-2K^s^	HβP		B10.S mice	34.1.2		

H-2K^b^	HβP	BALB/B mice	28.13.3

H-2D^b^	HβP, Hβ 2, H	BALB/B mice	28.14.8

H-2K^s^	HβP	Insulin receptor	NIH 3T3 HIR cells	R1-9.6	FRET by flow cytometry	Liegler et al. (1991)

H-2K^s^	2			CP28		

H-2D^q^, H-2L^q^	HβP	30.5.7

H-2D^q^, H-2L^q^	HβP, Hβ, H	28.14.8

H-2^b^	2	Insulin receptor	C57BL/6J mice	anti IRβ (IP) MCA51R (blot)	IP followed by SDS-PAGE	Dixon-Salazar et al. (2014)

HLA-A3	HβP, H 2	Insulin receptor 1	U937 cells	4F2 (IP), GAP-A3 (blot)	Cell surface labeling of IR with^125^I-photoreactive insulin analog followed by IP and SDS-PAGE	Due et al. (1986)

HLA-A1	2	Insulin receptor 3	LCL 721.13 cells	GSC142.1	Cell surface labeling of IR with^125^I-insulin, followed by cross-linking, IP, and SDS-PAGE	Reiland and Edidin (1993)

HLA-A2	HβP		LCL 721.1 cells	BB7.2		

HLA-A2	HβP		LCL 721.45.1 cells	BB7.2		

HLA-A2	HβP		LCL 721.53 cells	BB7.2		

HLA-A2	HβP	LCL 721.1 cells	PA2.1

**HLA-B5**	HβP	961 cells	anti β_2_m

HLA-B8	2	LCL 721.13 cells	B8

HLA-A2, A25	HβP, H	Insulin receptor 3	IM-9 cells	B8.1.23.2 B.9.12.1	Cell surface labeling of IR with^125^I- insulin analog followed by crosslinking, IP and SDS-PAGE	Samson et al. (1986)

B21, B22, Cw4	HβP

HLA-A2	H	Insulin receptor 1	Proteoliposomes	–	FRET by flow cytometry using proteoliposomes containing purified IR and HLA-A2	Ramalingam et al. (1997)

H-2K^k^, H-2D^k^	HβP	LH/hCG receptors	Ovaries of C3H mice	3.83 or 16.1.2	Homogenates incubated with^125^I-hCG followed by IP with anti-MHC mAbs and reprecipitation of LH/hCG receptors by polyethylene glycol	(Solano et al., 1988a), (Solano et al., 1988b)

H-2K^d^	HβP		Ovaries of BALB/c mice	34.1.2		

H-2D^d^	H	34.2.12

HLA-A, B, C	HβP	CD1a	Human thymic-derived cells	W6/32	Cell surface iodination (^125^I) followed by IP, SDS-PAGE and chymotryptic peptide mapping	Amiot et al. (1988)

HLA-A, B, C	HβP, H	A1.4

HLA-A, B, C	HβP	CD8	Human activated PBTC	anti-CD8	Cell surface iodination (^125^I) followed by IP, SDS-PAGE and chymotryptic peptide mapping	Bushkin et al. (1988)

HLA-A, B, C	HβP	W6/32

HLA-A, B, C	HβP	CD8 3	T cell clone, activated T cells	W6/32, BB7.7	Metabolically labelling of cells followed by cell surface cross-linking, IP, SDS-PAGE and chymotryptic peptide mapping	Blue et al. (1988)

HLA-A, B, C	H	CD8	Activated PBL	HC-10	Cell surface biotinylation followed by IP and SDS-PAGE	Santos et al. (2004)

H-2K^k^	HβP	CD8	KB5.C20 cells	Anti-CD8	Cell surface iodination (^125^I) followed by IP and SDS-PAGE	Auphan et al. (1991)

H-2K^k^	HβP	H-100-5/28
HLA-A66	HβP	IL-2 receptor	HUT-102B2 cells	W6/32	Metabolically labelling of cells followed by, IP, SDS-PAGE and proteolytic peptide mapping	Sharon et al. (1988)

HLA-A66	HβP	IL-2 receptor	HUT-102B2 cells	W6/32	FRET by flow cytometry	Szöllösi et al., (1987)

HLA-A, B, C	HβP	IL-2 receptor	Activated PBTC	W6/32	FRET by flow cytometry	Harel-Bellan et al. (1990)

HLA-A, B, C	HβP	IL-2 receptor	FT7.10 cells	W6/32	FRET by flow cytometry, apFRET	Mocsar et al. (2016)

HLA-A66	HβP	IL-2 receptor	HUT-102B2 cells	KE-2	FRAP	Edidin et al. (1988)

HLA-A, B, C	HβP	IL-15 receptor	FT7.10 cells	W6/32	FRET by flow cytometry, apFRET	Mocsar et al. (2016)

**H-2D**^**d**^	H	Ly49A	C1498 cells transfect- ed to express H-2D^d^	34.2.12	IP followed by SDS-PAGE	Doucey et al. (2004)

H-2D^b^	2	H141-30

**H-2D**^**d**^	2	Ly49A 3	C1498 cells transfect-ed to express HA-H 2D^d^ and VSV-Ly49A	anti-VSV (IP) anti-HA (blot)	Cell surface cross-linking followed by IP and SDS-PAGE	Back et al. (2009)

HLA-A3, B07, C07	HβP	HLA-DR	PGF cells	W6/32	FRET by flow cytometry	Szöllösi et al., (1989)

	HβP	HLA-DQw1,3		W6/32		

HβP	HLA-DQw1	W6/32				

HβP	HLA-DP	W6/32				

HLA-B7	HβP	HLA-DR		BB7.1		

HLA-B7	HβP	HLA-DQw1,3	BB7.1

HLA-B7	HβP	HLA-DP	BB7.1

HLA-A3	HβP	HLA-DR	GAP-A3

HLA-A3	HβP	HLA-DQw1,3	GAP-A3

HLA-A3	HβP	HLA-DP	GAP-A3

HLA-A3, B07, C07	HβP	HLA-DR	JY cells	W6/32	FRET, TEM, AFM	Jenei et al. (1997)

HLA-A66	HβP	HUT-102B2 cells	W6/32

HLA-A2	2	HLA-DQA	JY cells	–	Cross-linked MS	Armony et al. (2021)

HLA-A2	2	HLA-DRA		–		

HLA-A2	2	HLA-DRB		–		

HLA-B7	2	HLA-DRA		–		

HLA-B7	2	HLA-DRB	–

HLA-C7	2	HLA-DRA	–

HLA-C7	2	HLA-DRB	–

HLA-A3	H	HLA-F	PLH cells	anti HLA-F (IP), HCA2 (blot)	IP followed by SDS-PAGE	Goodridge et al. (2010)

HLA-A66	HβP	ICAM-1	HUT-102B2 cells	W6/32	Cell surface iodination (^125^I) followed by IP and SDS-PAGE	Szöllösi et al. (1987)

HLA-A2	2	ICAM-1	JY cells	PA2.1	Cell surface biotinylation followed by cross-linking, IP and SDS-PAGE	Lebedeva et al. (2004)

**Abbreviations**: AFM: atomic force microscopy; FRAP: fluorescence recovery after photobleaching; hCG: human chorionic gonadotrophin; MS: mass spectrometry; IP: immunoprecipitation; IR: insulin receptor; LH: luteinizing hormone; mAb: monoclonal antibody; PBTC: peripheral blood T cells; PBL: peripheral blood lymphocytes; apFRET: acceptor photobleaching FRET.; TEM: transmission electron microscopy.

**Notes:** First column shows endogenously expressed (not bold) and transfected (bold) allotypes by each cell line. A list of the allotypes expressed by cells and mice, the binding specificity, and the class I species (HβP, Hβ or H) that are recognized by antibodies can be found in Table 3, Table 4, respectively.

1 Associations were reduced after addition of exogenous β_2_m.

2 Uncertainty about the molecular species (HβP, Hβ and/or H) involved in the heterotypic association.

3 Chemical cross-linking of the cell surface is necessary to detect or enhance detection of interacting proteins.

**Table 3:** Human and mouse class I allotypes expressed by cell lines and mice.

Cell line/mouse strain	Expressed allotypes	Ref.
961	HLA-B5. Generated by stable HLA-B5 expression in the HLA negative LCL 721.221 cell line	(Reiland and Edidin, 1993; Shimizu and DeMars, 1989)

18.48	H-2L^d^	Capps et al. (1989)

C1498	NK T cell line derived from H-2^b^ mice	(Doucey et al., 2004; LaBelle and Truitt, 2002)

C58	Rat-derived cell line	Lynch et al. (2009)

CEM	HLA-A1, A31, B8, B40	Robinson et al. (2020)

EL4	H-2^b^	(Capps et al., 1989; Kane, 1994)

ELd3	H-2^b^, H-2L^d^ (an H-2L^d^ transfectant of EL4 cells)	(Capps et al., 1989, 1993; Zuniga et al., 1983)

FT7.10	HLA-A, B, C	Mocsar et al. (2016)

HCT-L^d^3	HLA negative, β_2_m negative, human cell line. Stably transfected to express H-2L^d^	(Capps et al., 1993; Gattoni-Celli et al., 1988)

HUT-102B2	HLA-A66	Robinson et al. (2020)

IM-9	HLA-A2, A25, B21, B22, Cw4	Samson et al. (1986)

Jeg-3	HLA-G	Gonen-Gross et al. (2005)

Jesthom	HLA-A2, B27, C1	Robinson et al. (2020)

Jurkat	HLA-A9, A25, B7, B41	Litwin et al. (1993)

JY	HLA-A2, B7, C7	Robinson et al. (2020)

KB5.C20	H-2^k^	Auphan et al. (1991)

KG-1	HLA-A30, A31, A33, B35	(Lynch et al., 2009; Koeffler et al., 1980)

LCL 721.1	HLA-A2, B5, C	(Reiland and Edidin, 1993; Kavathas et al., 1980)

LCL 721.13	HLA-A1, B8, C	(Reiland and Edidin, 1993; Kavathas et al., 1980)

LCL 721.221	HLA negative	Shimizu and DeMars (1989)

LCL 721.45.1	HLA-A2, B5, C	(Reiland and Edidin, 1993; DeMars et al., 1983)

LCL 721.53	HLA-A2, C	(Reiland and Edidin, 1993; Shimizu and DeMars, 1989)

_L_-K^b^	L929 mouse fibroblasts (H-2^k^) stably transfected to express H-2K^b^	(Lu et al., 2012; Lapham et al., 1993)

NIH 3T3 HIR	NIH 3T3 (H-2D^q^, H-2K^s^, H-2L^q^) stably transfected to express the human IR^a^	Liegler et al. (1991)

PGF	HLA-A3, B7, C7	Robinson et al. (2020)

PLH	HLA-A3, B47, C6	Robinson et al. (2020)

Raji	HLA-A3, B15, C3, C4	Robinson et al. (2020)

RE1	β_2_m negative, H-2 negative	(Verland et al., 1989; Bix and Raulet, 1992)

STF1	HLA-A3, B15, C14	(de la Salle et al., 1994; Brunnberg et al., 2021)

T2	HLA-A2, B5	Salter and Cresswell (1986)

U937	HLA-A3, A31, B18, B51, C1, C7	Gebreselassie et al. (2006)

AKR/J mice	H-2^k^	Chvatchko et al. (1983)

B10.S mice	H-2^s^	Phillips et al. (1986)

BALB/B mice	H-2^b^	Phillips et al. (1986)

BALB/c mice	H-2^d^	Solano et al. (1988a)

C3H mice	H-2^k^	Phillips et al. (1986)

C57BL/6 mice	H-2^b^	(Chvatchko et al., 1983; Fehlmann et al., 1985)

a IR: insulin receptor.

**Table 4:** Allotype and species (HβP, Hβ, H) specificities of antibodies to human and mouse class I molecules.

Antibody	Allotype	Recognized species	Ref.
11.4.1	H-2K^k^	HβP	(Fehlmann et al., 1985; Oi et al., 1978)

15.5.5S	H-2D^k^. Cross-reaction with H-2K^d^, H-2K^f^	^2^	Ozato and Sachs (1980)

16.1.2	H-2K^k^. Cross-reactions with H-2K^q^, H-2K^p^, H-2K^r^	HβP	(Solano et al., 1988a; Bix and Raulet, 1992; Ozato and Sachs, 1980)

16.3.1	H-2K^k^. Cross-reactions with H-2K^q^, H-2K^p^, K^r^	HβP	(Fehlmann et al., 1985; Ozato and Sachs, 1980; Lemke et al., 1979)

20.8.4	H-2D^b^, H-2 K^b^	HβP	(Fehlmann et al., 1985; Ozato and Sachs, 1981)

25-D1.16	SIINFEKL-pulsed H-2K^b^cells	HβP	(Porgador et al., 1997; Mareeva et al., 2008)

28.13.3	H-2K^b^	HβP	Ozato and Sachs (1981)

28.14.8	H-2L^d^, H-2L^q^, H-2D^b^, H-2D^q^	HβP, Hβ and H	(Lie et al., 1991; Townsend et al., 1990; Allen et al., 1986b; Fraser et al., 1987)

3.83	H-2D^k^, H-2K^k^. Cross-reaction with H-2K^b^, H-2K^s^, H-2K^q^, H-2K^p^, H-2K^r^	HβP	Ozato and Sachs (1980)

30.5.7	H-2L^d^, H-2D^q^. Cross-reactions with H-2L^q^	HβP	(Liegler et al., 1991; Lie et al., 1991; Harris et al., 1998)

34.1.2	H-2K^d^. Cross-reaction with H-2K^b^, H-2K^s^, H-2K^q^, H-2K^p^, H-2K^r^	HβP	(Phillips et al., 1986; Solano et al., 1988a)

34.2.12	H-2D^d^	H	(Solano et al., 1988a; K et al., 1982; Thor et al., 1993)

34.4.20	H-2K^b^	HβP	Catipovi ć et al. (1992)

4F2	α chain of the IR a	–	(Due et al., 1986; Haynes et al., 1981; Srikanta et al., 1987)

64.3.7	H-2L^d^, H-2L^q^, H-2K^d^	H	(Lie et al., 1991; Shiroishi et al., 1985; Simone et al., 2012)

87G	HLA-G1	HβP	Menier et al. (2003)

A1.4	HLA-A, B, C	HβP and H	(Bushkin et al., 1986, 1988)

AF6-88.5	H-2K^b^	HβP	Rock et al. (1991b)

B22.249	H-2D^b^	HβP	(Capps et al., 1993; Potter et al., 1984; Ferez et al., 2014; Allen et al., 1986b)

B8	HLA-B8	b	Reiland and Edidin (1993)

B8.1.23.2	HLA-A, B, C	HβP and H	Reba ï and Malissen (1983)

B9.12.1	HLA-A, B, C	HβP	Reba ï and Malissen (1983)

BB7.1	HLA-B7	HβP	Brodsky et al. (1979a)

BB7.2	HLA-A2, A28 c	HβP	(Brodsky et al., 1979a; Parham and Brodsky, 1981)

BB7.7	HLA-A, B, C	HβP	Brodsky et al. (1979a)

BBM.1	β_2_m (free β_2_m and non-covalently linked β_2_m to HLA molecules)	HβP	Brodsky et al. (1979b)

CP28	H-2D^d^, D^s^. Cross-reaction with K^s^	HβP?	(Liegler et al., 1991; Linsk et al., 1989; Philipps et al., 1985)

G233	HLA-G	HβP	Loke et al. (1997)

GAP-A3	HLA-A3	HβP	Berger et al. (1982)

GSC142.1	HLA-A1	b	Reiland and Edidin (1993)

H-100-30/23	H-2D^k^, H-2K^k^	HβP?	(Fehlmann et al., 1985; Lemke et al., 1979)
H-100-5/28	H-2K^k^	HβP	(Fehlmann et al., 1985; Auphan et al., 1991; Lemke et al., 1979)

H141-30	H-2D^b^	b	Lemke et al. (1979)

HC-10	HLA-B. Cross-reaction with HLA-C and HLA-A	H	(Stam et al., 1986, 1990; Baas et al., 1992)

HCA2	HLA-A	H	(Stam et al., 1990; Baas et al., 1992)

KE-2	HLA-A, B, C	HβP	Damjanovich et al. (1995)

MCA51R	H-2^b^	HβP b	(Dixon-Salazar et al., 2014; Fukumoto et al., 1982)

ME1	HLA-B7, B27, B22	HβP	(Ellis et al., 1982; Malik et al., 1999)

MEM-G/09	HLA-G1	HβP	Menier et al. (2003)

MEM-G/11	HLA-G	HβP	Boyson et al. (2002)

PA2.1	HLA-A2	b	(Brodsky et al., 1979a; Parham and Bodmer, 1978)

PA2.6	HLA-A, B, C	HβP	Brodsky et al. (1979a)

R1-9.6	H-2K^b^, H-2K^k^, H-2K^s^, H-2D^b^, H-2D^d^, H-2D^f^, H-2L^d^	HβP	(Liegler et al., 1991; Koch et al., 1983)

W6/32	HLA-A, B, C	HβP	(Brodsky et al., 1979a; Barnstable et al., 1978; Ladasky et al., 1999)

a IR: insulin receptor.

b Uncertainty about the molecular species (HβP, Hβ and/or H) recognized by the antibody.

c A specific variant of HLA-A*28 is recognized by BB7.2.

## 2 MHC class I *in cis* homotypic associations

Homotypic associations were first described both for human (Matko et al., 1994; Chakrabarti et al., 1992) and murine (Capps et al., 1993) class I molecules in the early 1990s, and many times since then (Table 1). The first reports stated that β_2_m-free heavy chains (H, Fig. 1A) are necessary participants of homotypic associations between class I molecules, since such associations were only detected when class I species reactive with the mAb HC-10 (*i.e.,* H; Table 4) were also present at the cell surface (Matko et al., 1994; Chakrabarti et al., 1992). Also, class I associations were reduced after addition of exogenous β_2_m, which bound to H at the cell surface (Capps et al., 1993; Chakrabarti et al., 1992; Bodnar et al., 2003).

Depending on the MHC I allotype (Table 3) and on the specificity of the antibodies (Table 4), two class I molecular species were mainly found to be involved in dimer formation, giving rise to three non-covalent homotypic complexes, namely HβP/HβP, HβP/H, and H/H (Fig. 1B and Table 1), and to three covalently linked complexes, HβP-HβP, H–H (both linked through extracellular domains) and H**.**H (linked through cytoplasmic domains) (Fig. 1C and Table 1). Still, some conformation-specific monoclonal antibodies that are commonly thought to depend on the presence of peptide, such as W6/32, might also bind to Hβ species of some allotypes, and thus, the participation of, or a requirement for, Hβ in these complexes cannot be excluded. Higher-order associations such as trimers, tetramers, and oligomers containing approximately 20–250 class I molecules were also described (Lu et al., 2012; Matko et al., 1994; Capps et al., 1993; Bodnar et al., 2003; Triantafilou et al., 2000; Ferez et al., 2014; Blumenthal et al., 2016; Fooksman et al., 2006; Jenei et al., 1997). The size of class I oligomeric associations at steady state depends on both the rates of class I entry into and exit from these associations, the latter being partially regulated by components of the actin cytoskeleton (Blumenthal et al., 2016; Lavi et al., 2012). This dynamic equilibrium defines the lifetime of associations at the plasma membrane, which were described to be in the range of seconds (Blumenthal et al., 2016; Lavi et al., 2007, 2012), minutes (Matko et al., 1994), and even hours (Lu et al., 2012), possibly depending on the allotype and/or the cell type. The rate of association is also related to plasma membrane cholesterol levels, although the molecular mechanism by which cholesterol influences the size and dynamics of class I associations is unclear (Ferez et al., 2014; Bodnár et al., 1996).

Even though the existence of homotypic associations between class I molecules has been recognized for almost half a century, the molecular mechanisms governing the interactions have only recently begun to emerge. Dimerization mediated by the formation of disulfide bonds between cysteine residues located in the extracellular domain of some allotypes has been described, including HLA-B27 through Cys-67 (Allen et al., 1999) (Fig. 1C, H-H), or Cys-42 in the nonclassical HLA-G (Boyson et al., 2002; Gonen-Gross et al., 2003, 2005; Shiroishi et al., 2006) (Fig. 1C, HβP-HβP). Relatively recently, a different type of covalent association - through cysteine residues localized in the cytosolic domain of class I - has been described (Lynch et al., 2009) (Fig. 1C, H**.**H). In HLA-B27, the cytosolic disulfide bond occurs between Cys-325, and in HLA-A2, it probably involves its unique Cys-339, since deletion of the cytosolic domain prevented dimerization. Covalent association through the cytosolic tail was found in exosomes derived from various cell lines, but it was rarely found in the corresponding live cells (Lynch et al., 2009; Makhadiyeva et al., 2012), where the cytosol maintains a strong reducing environment. Indeed, the glutathione concentration in exosomes is only a quarter of that in the cytosol (Lynch et al., 2009), and conditions that deplete intracellular glutathione (such as treatment with oxidizers (Makhadiyeva et al., 2012) or changes in cell density and proliferation rate (Baia et al., 2016)) resulted in class I cytosolic-mediated dimer formation in live cells. In one study, HLA-A2 disulfide-bonded dimers were shown to be an artifact of sample processing, which could be avoided by the inclusion of iodoacetamide (a blocker of disulfide bond formation through covalent modification of free cysteines) (Boyson et al., 2002). Thus, current evidence limits the formation of covalent class I dimers through cytosolic cysteine residues (Fig. 1C, H.H) to the specific oxidizing conditions found in exosomes.

In contrast to the covalent dimers, disulfide bonds – at least those observed so far – do not suffice to explain the observations of class I trimers and oligomers. Purely disulfide-mediated association would mostly limit class I complexes to dimers, since the formation of higher order oligomers would require additional non-covalent interactions. Another line of evidence supporting a non-covalent nature of associations is that some class I dimers were temperature-sensitive. Heating of immunoprecipitated samples containing HLA dimers and trimers to temperatures above 37 °C abolished their association, suggesting that non-covalent forces govern the dimerization (Triantafilou et al., 2000).

At this stage of research into class I homotypic associations, conflicting descriptions of the phenomena still exist. The human lymphoblastoid cell line JY has been extensively used in the research of non-covalent class I associations, since it displays a high degree of class I oligomerization as well as substantial levels of H on its plasma membrane. However, under similar experimental conditions in the same cell line, some reports observed dimers and oligomers of class I molecules composed of HβP/HβP or HβP/H on the surface of JY cells (Matko et al., 1994; Chakrabarti et al., 1992; Bodnar et al., 2003), whereas others only described the formation of HβP/H but not of HβP/HβP (Bodnár et al., 1996) or were unable to detect any homotypic class I associations at all (Szöllösi et al., 1989). Recently, mass spectrometry was used to map the native HLA interactome on the plasma membrane of JY cells (Armony et al., 2021). This work showed non-covalent interactions between class I molecules in detail, but it was not established which species of class I were part of the associations. Similarly, murine class I H-2D^b^ molecules were found to form non-covalent oligomers containing HβP species (Ferez et al., 2014), but under different experimental conditions, only H**.**H covalent dimers were detected (Capps et al., 1993).

Human class I allotypes are able to form HβP/HβP dimers (Ferez et al., 2014; Jenei et al., 1997; Lynch et al., 2009). For murine allotypes, these dimers were only reported for H-2K^b^ after expression of its extracellular domain fused to a C-terminal histidine tag. Capture though a nickel chelating lipid then allowed binding of the protein to a lipid bilayer and mimicking the normal in vivo display (Mitra et al., 2004). In living cells, HβP of H-2K^b^ were detected in class I clusters (Lu et al., 2012); however, it is not clear whether they interact directly with each other. Recently, a novel two-hybrid assay that uses antibody micropatterns printed on glass to capture cell surface class I molecules (Dirscherl et al., 2017) was used to study the formation of H-2K^b^ associations at the plasma membrane of living cells (Dirscherl and Springer, 2018). Briefly, cells express two different tagged versions of the same class I molecule, namely an N-terminally (extracellularly) HA-tagged version of H-2K^b^ (*i.e.*, HA–H-2K^b^), and a C-terminally (intracellularly) GFP-tagged variant (H-2K^b^–GFP). Cells are then seeded on a surface covered with anti-HA antibodies that are arranged in a micropattern. Cell surface HA–H-2K^b^ is captured by the antibodies in the micropatterns. If a homotypic interaction between H-2K^b^–GFP and HA–H-2K^b^ occurs, then GFP patterns can be detected by fluorescence microscopy. When using a TAP-deficient cell line and manipulating the incubation conditions (temperature, exogenous addition of class I binding peptide), this assay also reveals the species of the interacting molecules (HβP, Hβ or H). The STF1 cell line lacks the transporter associated with antigen processing (TAP) and cannot load MHC I proteins with high-affinity peptides in the ER (de la Salle et al., 1994). This feature allows the accumulation of Hβ at the cell surface by incubation at 25 °C. The rapid loss of β_2_m after incubation at 37 °C results in the formation of free heavy chains (H) and can be prevented by the exogenous addition of class I binding peptide to accumulate HβP at the cell surface. Results using this approach have provided conclusive evidence for the existence of non-covalent H-2K^b^ associations at the plasma membrane of living cells mainly consisting of H, whereas HβP does not associate (Fig. 1B, H/H) (Dirscherl et al., 2017, 2018). This procedure might be extended to other class I allotypes.

## 3 Biological roles of MHC class I homotypic associations

Ascribing functional roles to associations of class I molecules has always been a major challenge, but some functional evidence is now emerging. One idea is that the formation of higher-order oligomers of HβP of classical class I molecules (HLA-A, -B, and –C) might constitute a more effective way of presenting antigens to T cells (Matko et al., 1994; Chakrabarti et al., 1992). Such (HβP)_n_ in the membrane of target cells might provide T cells with local areas that display multiple copies of the antigenic peptide. Oligomers have decreased rotational and lateral mobility that may facilitate T cell recognition by reducing scanning times (Fooksman et al., 2006), and they might promote the formation of a more stable immunological synapse with stronger T cell activation signals (Bodnar et al., 2003). This model of interaction can help explain the high sensitivity of T cell responses despite the low affinity of the receptor–ligand interactions involved (Ferez et al., 2014). Finally, organization of class I molecules as oligomers in the plasma membrane might be responsible for the enhancement of T cell responses against tumors where low-abundance peptides are expressed among an ocean of self-peptides (Lu et al., 2012; Fooksman et al., 2006).

Formation of free heavy chain dimers (H/H) might also play a role in sequestering this species to prevent re-binding of β_2_m and exogenous peptides, avoiding innocent bystander killing by cytotoxic T cells (Capps et al., 1993). Recognition of surface HLA-B27 dimers (H–H) by NK cell immunoreceptors may contribute to the pathogenesis of autoimmune inflammatory disorders such as ankylosing spondylitis (AS) (Chen et al., 2017; Bird et al., 2003). There is still much to learn though, since some HLA-B27 subtypes, for example B*27:05, are statistically associated with the development of AS, but others, even closely related and also dimer-forming, such as B*27:09, are not (Bird et al., 2003; Benjamin et al., 1991; Tsai et al., 2002; Raine et al., 2006). Other disease-associated class I molecules have not been thoroughly tested for dimerization (McGonagle et al., 2015; Arosa et al., 2021).

Physiologically, the non-classical MHC class I (or class Ib) molecule HLA-G is exclusively expressed in trophoblast cells that invade the maternal endometrium during the formation of the placenta (Kovats et al., 1990). HLA-G forms disulfide-linked HβP dimers, trimers, and oligomers on the cell surface of transfected cell lines (Boyson et al., 2002; Gonen-Gross et al., 2003) and on normal first-trimester trophoblast cells (Apps et al., 2007). These HβP-HβP complexes were found to bind to the leukocyte immunoglobulin-like inhibitory receptor 1 (LILR1) (Gonen-Gross et al., 2003, 2005; Shiroishi et al., 2006) in decidual NK cells with a nanomolar dissociation constant, as opposed to micromolar for monomeric HLA-G (Shiroishi et al., 2006). These results are in line with the high local concentrations of HLA-G that are necessary to trigger LILR1-mediated inhibition (Chapman et al., 1999). Also, HLA-G HβP-HβP dimers on the trophoblast cell surface engage with LILR1 on decidual macrophages, inducing the local production of anti-inflammatory cytokines (Apps et al., 2007). On the other hand, HLA-G homodimers were also proposed to stimulate decidual macrophages and NK cells through the engagement of LILR1 and KIR2DL3 (killer cell immunoglobulin-like receptor 2DL3), respectively, to secrete proinflammatory cytokines that might play a role in the implantation of the developing embryo (Li et al., 2009). In contrast to these HβP-HβP dimers, there are also H–H dimers and mixed complexes of HβP and H of HLA-G on the trophoblast cell surface, but these cannot engage the NK cell receptors and may even interfere with this interaction (Gonen-Gross et al., 2005). Thus, the enhanced binding of HLA-G HβP-HβP homodimers to NK and macrophage cell surface receptors possibly contributes to the establishment of a tolerogenic maternal-fetal interface in which a delicate balance between pro-inflammatory and anti-inflammatory signals exists. In this way, immune cells are able to discriminate between the fetally derived trophoblast cells that express HLA-G, and the maternal cells that express classic HLA-I molecules.

In summary, although there is still a long way to go towards a complete understanding of the biological roles of MHC class I homotypic associations, current evidence points to important functions of these complexes in physiology and disease. This encourages further research in the identification of the molecular mechanisms that mediate the formation and occurrence of these associations, which will undoubtedly lead to a better understanding of their cellular functions, and the potential development of new strategies to deal with diseases.

## 4 MHC class I *in cis* heterotypic associations

At the plasma membrane, MHC class I molecules associate not only with class I molecules. Several studies have also described associations with other cell surface proteins (Table 2). Although these studies provide evidence for diverse roles for class I molecules in non-immunological processes, the exact functions of these associations and their molecular mechanisms are difficult to define due to the different experimental approaches and biological models employed. In the following sections, we present an overview of most proteins that were found to interact with class I molecules *in cis* at the cell surface, and we discuss the evidence that supports biological roles for these heterotypic associations.

The first characterized interaction partner of class I molecules was the insulin receptor (IR), which associates with several mouse (Dixon-Salazar et al., 2014; Chvatchko et al., 1983; Fehlmann et al., 1985; Verland et al., 1989; Phillips et al., 1986; Liegler et al., 1991) and human allotypes (Due et al., 1986; Ramalingam et al., 1997; Reiland and Edidin, 1993). Direct evidence of interactions with the luteinizing hormone receptor also exists (Solano et al., 1988a, 1988b), and other studies exploited receptor-ligand binding experiments to provide indirect evidence for class I association with the epidermal growth factor receptor (Schreiber et al., 1984), the receptor for γ-endorphins (Claas et al., 1986; Mommaas et al., 1991), cholinergic receptors (Cremaschi et al., 1992), and β-adrenergic receptors (Cremaschi et al., 1994). Heterotypic interactions between classical class I molecules and cell surface proteins are not restricted to hormone and neurotransmitter receptors, since *in cis* interactions with various HLA class II proteins (Jenei et al., 1997; Szöllösi et al., 1989; Armony et al., 2021), the lipid-presenting molecule CD1a (Amiot et al., 1988), the coreceptor CD8 (Bushkin et al., 1988; Blue et al., 1988; Santos et al., 2004; Auphan et al., 1991), immune receptors for IL-2 (Sharon et al., 1988; Szöllösi et al., 1987; Harel-Bellan et al., 1990; Mocsar et al., 2016; Edidin et al., 1988), IL-15 (Mocsar et al., 2016), Ly49 (Doucey et al., 2004; Back et al., 2009), the non-classical class I molecule HLA-F (Goodridge et al., 2010), and even the adhesion molecule ICAM-1 (Szöllösi et al., 1987; Lebedeva et al., 2004) have all been reported. These might play a role in regulating the immune response as discussed in the next section.

Similar to the homotypic interactions, associations of class I molecules with the IR were demonstrated to be dependent, at least in part, upon the presence of H species, since incubation with an excess of β_2_m caused a reduction in the amount of HLA/IR complexes (Due et al., 1986; Ramalingam et al., 1997). These observations also imply that reversible non-covalent associations are involved. Other reports have supported the non-covalent nature of the interaction between class I molecules and CD1a (Amiot et al., 1988) or CD8 (Bushkin et al., 1988; Blue et al., 1988; Auphan et al., 1991). Further analyses indicated that *in cis* association between CD8 and class I is also, at least in part, mediated by disulfide bonding, which might be susceptible to cleavage during the processing of cell lysates (Blue et al., 1988). Chemical cross-linking was necessary in some cases to detect detergent-labile interactions between class I and IR (Fehlmann et al., 1985; Reiland and Edidin, 1993; Samson et al., 1986), CD8 (Bushkin et al., 1988; Blue et al., 1988) or Ly49A (Back et al., 2009), which also suggests that non-covalent associations are involved. The *in cis* interaction with the co-receptor CD8 was dependent on the presence of H species, since higher levels of CD8 were co-precipitated when the class I species recognized by HC-10 mAb (i.e., H; Table 4) also increased (Santos et al., 2004).

## 5 Biological roles of MHC class I heterotypic associations

The initial demonstration of a physical interaction between class I molecules and the insulin receptor (IR) has prompted researchers to re-evaluate the physiological functions of class I molecules in the context of non-immunological processes (Chvatchko et al., 1983) such as signal transduction and the control of IR abundance and function (Fehlmann et al., 1985; Verland et al., 1989). With the subsequent findings of their complexes with other cell surface proteins (Szöllösi et al., 1989; Solano et al., 1988a; Solano et al., 1988b; Amiot et al., 1988; Bushkin et al., 1988; Blue et al., 1988; Sharon et al., 1988; Szöllösi et al., 1987; Edidin et al., 1988), the idea that class I molecules regulate the activity not only of IR but also of different membrane receptors began to gain momentum (Verland et al., 1989). The expression of mouse class I affected insulin binding to the IR in an allotype-dependent fashion: one class I allotype (H-2D^b^) was correlated with higher insulin binding affinity in mouse (Verland et al., 1989), whereas expression of H-2K^s^, H-2D^q^, or H-2L^q^ did not affect the binding affinity of the IR for its ligand but caused a reduction in the rate of insulin-stimulated endocytosis of the receptor (Liegler et al., 1991). In the case of human class I molecules, both HLA-A2 and A3 allotypes interact with the IR, and the affinity of insulin increased when these HLA molecules where present at the plasma membrane (Due et al., 1986; Ramalingam et al., 1997). These results were in line with another report that described coprecipitation of high-affinity IR with HLA-A2 from an HLA-A^+^B^−^ cell line (Reiland and Edidin, 1993). The same amount of IR coprecipitated with HLA-A2 from an HLA-A^+^B^+^ cell line, but these two cell lines show remarkable differences in the insulin binding affinity of their cell surface IR. While the HLA-A^+^B^−^ cell line expresses high-affinity IR, HLA-A^+^B^+^ cells have low-affinity IR, and thus, the amount of class I molecules bound to the IR does not define its affinity for insulin (Reiland and Edidin, 1993). Rather, each allotype might differentially affect the receptor by either inducing conformational changes and/or even forming part of the receptor complex (Due et al., 1986; Reiland and Edidin, 1993). In agreement with this hypothesis, the affinity of insulin binding correlates with the expression of particular HLA allotypes and is similar in cell lines that express the same HLA class I molecules (Kittur et al., 1987). The binding of insulin to the HLA-negative cell line 721.221 indicates that class I molecules are not obligate subunits of the functional IR, but they can affect the quality and affinity of insulin binding sites when expressed with the receptor (Kittur et al., 1987).

In addition, another interesting although not completely explored mechanism is the co-regulation by two or more allotypes binding to a receptor at the same time. The formation of such complexes has been experimentally demonstrated in mice, where the entire fraction of IR bound to H-2K^k^ was also associated with H-2D^k^ and vice versa (Phillips et al., 1986). For human class I, two or more HLA molecules were proposed to interact with the same IR (Reiland and Edidin, 1993). What such associations might look like, and how they influence the binding and activity of receptors, is not known.

The species of class I that binds to the IR is unknown. Still, indirect evidence suggests that the IR competes with β_2_m for binding to H. Furthermore, polymorphisms in the class I region involved in the association with β_2_m dictate the stability of the Hβ association (Ribaudo and Margulies, 1995). These data suggest H/IR complexes, and thus, class I molecules with low and high affinities for β_2_m might differentially associate with the IR and modulate its properties and functions. In contrast to this finding, β_2_m-associated class I molecules have also been found as part of HLA/IR complexes (Reiland and Edidin, 1993).

With respect to the functional regulation of IR by class I, IR tyrosine kinase activity increased with the class I/IR ratio, *i.e.*, as the amount of HLA molecules at the plasma membrane increased (Ramalingam et al., 1997). Tyrosine phosphorylation of HLA-A2 molecules also increased after binding to the IR, which enabled the subsequent binding of the downstream signaling-related molecule PI-3 kinase, implying that class I molecules are involved in the IR signaling cascade (Ramalingam et al., 1997). In the mouse brain, however, class I/IR association prevented signaling and led to a negative regulation of the number of synaptic connections. At the same time, neither expression nor trafficking of the IR were affected by its interaction with class I molecules (Dixon-Salazar et al., 2014). H species of HLA were also found to physically associate with CD8 and Lck kinase, and although no specific role was described, it might be interesting to assess if class I can modulate signaling events delivered by the CD8-Lck complex (Santos et al., 2004).

Class I can interact with NK cell receptors both *in trans* and *in cis*, using the same binding site beneath the peptide binding groove (Doucey et al., 2004; Back et al., 2009). The *in cis* interaction might then regulate the activity of the NK cell receptors. For example, expression of H-2D^d^ on NK cells of H-2^b^ origin led to a weaker Ly49A-mediated inhibition response, since *in cis* binding of H-2D^d^ to Ly49A association reduces the availability of Ly49A to associate *in trans* with H-2D^d^, its ligand on target cells (Doucey et al., 2004; Back et al., 2007).

Finally, *in cis* associations of class I molecules with ICAM-1 were proposed to enhance the formation of the immunological synapse through the accumulation of HLA-A2 and ICAM-1 molecules in plasma membrane regions were antigen presentation occurs (Lebedeva et al., 2004).

Altogether, despite partly contradictory reports and a substantially incomplete understanding of the molecular mechanisms, the evidence suggests several functional roles for class I molecules in the regulation of cell surface receptors at different levels.

## 6 Concluding remarks and future perspectives

Over the past four decades, many groups have provided evidence for the existence of complexes formed by class I molecules with other class I molecules (homotypic) or with other cell surface receptors (heterotypic). Compared to other advances in the class I field, understanding of these phenomena and their physiological role has grown slowly, but evidence supporting both immunological and non-immunological roles is accumulating. To conclude this review, we would like to formulate four challenges to the field.

First, for more detailed understanding of the associations, it will be crucial to establish which species of class I (HβP, Hβ, H) are involved in them, since class I homodimers might have different and even opposite functions depending on the species in the complex. One example for this is HLA-G, where HβP-HβP dimers are involved in the development of an immunotolerant environment during pregnancy (Gonen-Gross et al., 2003, 2005; Shiroishi et al., 2006), whereas mixed dimers composed of HβP and H and also H–H dimers seem to interfere with this process (Gonen-Gross et al., 2005).

Second, it will be important to standardize our observations by generating agreement between the differing results generated from different cell lines and from different class I allotypes. Such differing results may be real, or else they may be caused by technical variations in the experiments. This is especially true for non-covalent associations, which are more sensitive to disruption than disulfide-linked dimers when extracted from cell membranes. The use of detergents in the immunoprecipitation buffers can affect and even preclude the detection of non-covalent associations, and thus, very gentle detergents such as digitonin, otherwise known from peptide loading complex co-immunoprecipitations, might be required (Capps et al., 1993; Auphan et al., 1991). Addition of chemical crosslinkers prior to solubilization, although not always necessary (Dirscherl et al., 2018; Santos et al., 2004), has helped the detection of class I associations (Fehlmann et al., 1985; Samson et al., 1986), whereas in most studies, complexes were only observed in cross-linked samples (Reiland and Edidin, 1993; Bushkin et al., 1988; Blue et al., 1988; Back et al., 2009). Artifactual post-lysis associations might arise when membranes are solubilized for immunoprecipitation experiments (Boyson et al., 2002), and thus, the detection of interactions in the native plasma membrane environment of live cells is principally preferable.

Importantly, some methods might not detect class I molecules which are indirectly associated, *i.e.*, when another partner is involved in the interaction. Immunoprecipitation experiments have shown formation of such complexes between the insulin receptor (IR) and several class I molecules in mice, H-2D^k,b^ and H-2K^b,k,s^ (Phillips et al., 1986), since the entirety of IR bound to one allotype was also associated with the other. A live cell approach by FRET microscopy, on the other hand, did not detect associations between H-2D^q^ and H-2K^s^ molecules (Liegler et al., 1991). Discrepancy in results might arise by the limitation of the live cell approach to detect class I molecules that are part of a complex in which the IR acts as mediator in the interaction (H–2K/IR/H-2D, indirect association) and therefore, class I would be more than 10 nm apart from one another, which precludes energy transfer between fluorophores. Other possibilities are inappropriate orientation of the fluorophores that impedes energy transfer, that the H–2K/IR/H-2D complex is a post-lysis artifact of the immunoprecipitations, or that H-2D^q^ does not interact with H-2K^s^.

Indeed, the observation of the H–2K/IR/H-2D complex leads to the question whether other proteins are necessary for the formation of homotypic class I associations, *i.e.*, whether the participation of a non-class I protein might be required for the establishment of interactions between two or more class I molecules. The identification of such mediators will need proteomic screening methods. Recently, it has been possible to map the class I interactome at the plasma membrane via extracellular crosslinking followed by mass spectrometry (Armony et al., 2021), and these experiments did detect direct class I/class I homotypic associations. But such direct interactions between class I molecules do not necessarily prove that other proteins are absent from the complex; they may still be required for, and directly involved in, the formation of class I homotypic associations. Interestingly, to the best of our knowledge, the role of cytosolic proteins in the homotypic association of class I molecules has never been investigated.

Such technical insufficiency might obscure the difference between homotypic and heterotypic associations, since a H–2K/IR/H-2D complex might look like an H–2K/H-2D complex, if the presence of the IR is not specifically investigated. This is why, as a third challenge, it is desirable to come to a complete characterization of the constituents of class I associations, perhaps with a combination of genetic and proteomic (mass spectrometry) means and novel cell biological approaches. One example of a new method to detect interactions in the native plasma membrane environment of live cells and over a wider range of distances is a recently developed approach that employs antibody micropatterns printed on glass to capture cell surface class I molecules (Dirscherl et al., 2017), which was used to detect *in cis* class I homotypic associations in the plasma membrane of living cells and to define which class I molecular species (HβP, Hβ or H; Fig. 1A) they consisted of (Dirscherl et al., 2018). The approach is versatile and can be combined with other methods. It was recently used in combination with fluorescence recovery after photobleaching (FRAP) and single molecule co-tracking to study the stoichiometry and dynamics of H/H complexes (Dirscherl et al., 2022).

As a fourth, but not least, challenge, the exploration of the physiological role and meaning of homotypic and heterotypic class I associations is essential. Again, novel techniques will prove valuable. For example, the above micropatterning approach can be combined with the co-expression of proteins known to function as signaling-adaptor molecules or involved in early endocytic events to study the pathways that become active after the association took place. With this knowledge in hand, we can finally hope to understand how homotypic and heterotypic associations involving class I molecules help maintain health and/or contribute to disease pathogenesis and hopefully translate this knowledge to clinical medicine.

## Funding

This research was funded by the 10.13039/501100001659Deutsche Forschungsgemeinschaft, grant Sp583/18–1 to S.Sp.

## CRediT authorship contribution statement

**Fernando M. Ruggiero:** Investigation, Resources, Data curation, Writing – original draft, Writing – review & editing, Visualization. **Sebastian Springer:** Conceptualization, Methodology, Software, Validation, Formal analysis, Writing – original draft, Writing – review & editing, Visualization, Supervision, Project administration, Funding acquisition.

## Declaration of competing interest

The authors declare that they have no known competing financial interests or personal relationships that could have appeared to influence the work reported in this paper.
